# Flexibility in the Insulin Receptor Ectodomain Enables Docking of Insulin in Crystallographic Conformation Observed in a Hormone-Bound Microreceptor

**DOI:** 10.3390/membranes4040730

**Published:** 2014-10-10

**Authors:** Harish Vashisth

**Keywords:** insulin, insulin receptor, receptor tyrosine kinases, membrane receptors, docking, molecular dynamics simulations, signal transduction

## Abstract

Insulin binding to the insulin receptor (IR) is the first key step in initiating downstream signaling cascades for glucose homeostasis in higher organisms. The molecular details of insulin recognition by IR are not yet completely understood, but a picture of hormone/receptor interactions at one of the epitopes (Site 1) is beginning to emerge from recent structural evidence. However, insulin-bound structures of truncated IR suggest that crystallographic conformation of insulin cannot be accommodated in the full IR ectodomain due to steric overlap of insulin with the first two type III fibronectin domains (F1 and F2), which are contributed to the insulin binding-pocket by the second subunit in the IR homodimer. A conformational change in the F1-F2 pair has thus been suggested. In this work, we present an all-atom structural model of complex of insulin and the IR ectodomain, where no structural overlap of insulin with the receptor domains (F1 and F2) is observed. This structural model was arrived at by flexibly fitting parts of our earlier insulin/IR all-atom model into the simulated density maps of crystallized constructs combined with conformational sampling from apo-IR solution conformations. Importantly, our experimentally-consistent model helps rationalize yet unresolved Site 2 contacts of hormone with IR, and suggests ligand cross-linking of receptor subunits.

## 1. Introduction

**Figure 1:**
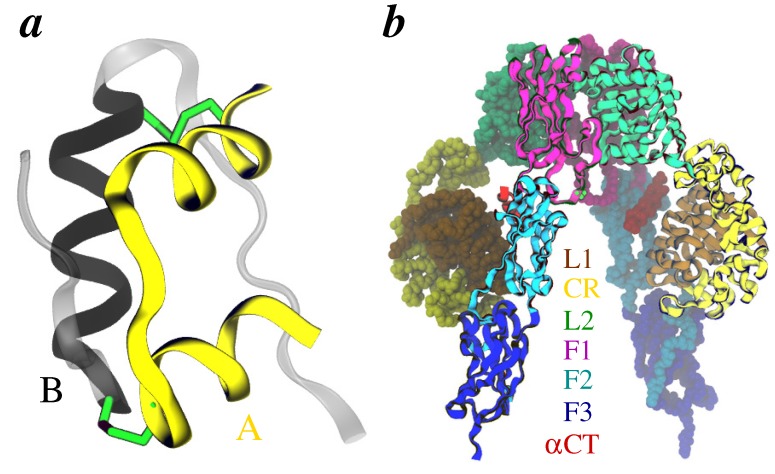
Three-dimensional folds of insulin and the IR ectodomain. **(**a**)** Helices in individual chains of hormone (PDB code 4INS) are rendered as yellow (A-chain) and black (B-chain) cartoons, while the flexible N- and C-termini of B-chain are transparent cartoons. **(**b**)** IR ectodomain homodimer (PDB code 3LOH) is shown with domains of one subunit as ribbons, while identical domains of the other subunit as space-filling. Individual domains are uniquely colored and labeled (*α*CT stands for the helical C-terminal segment of each *α*-chain of IR). Two interchain disulfide bonds in insulin and one in the receptor are shown in green sticks. In all panels, only backbone atoms are rendered for clarity.

Insulin is a therapeutically significant protein hormone secreted by the pancreatic *β* cells [1] that binds to the extracellular domains of the insulin receptor (IR) and activates the intracellular kinase domains. Mature forms of hormone and the receptor are comprised of two polypeptide chains: (a) the A- and B-chain (totaling 51 residues) in insulin; and (b) the *α*- and *β*-chains (totaling over 1300 residues) in IR [2,3,4,5]. Structurally, both chains of insulin are linked by two interchain disulfide bonds and have three *α*-helices (Figure 1a) with some flexible residues in the N- and C-termini of B-chain (B1-B8 and B21-B30) [6], while IR ectodomain has a homodimeric *∧*-shaped topology [7,8] where each subunit is comprised of a leucine-cysteine-leucine-rich (L1-CR-L2) motif [9] followed by three fibronectin repeats (F1-F2-F3) (Figure 1b) that in the *holo*-receptor are connected to an intracellular kinase domain via a single transmembrane helix [10,11,12,13,14,15,16,17,18,19]. The homologous type 1 insulin-like growth factor receptor (IGF1R) [20] shares similar topology [21].

**Figure 2:**
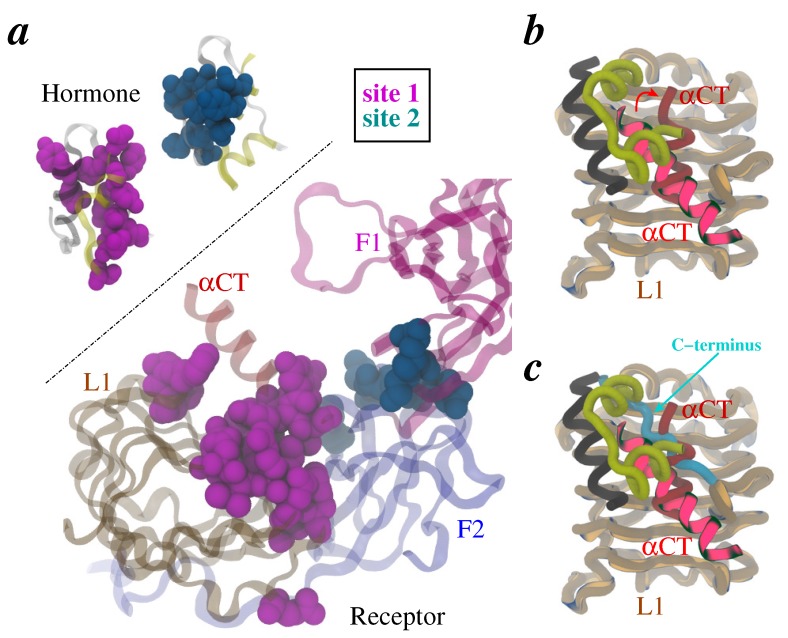
Insulin/IR binding interfaces. **(**a**)** Patches of residues that form Site 1 (magenta) and Site 2 (dark blue) on hormone and the receptor are shown as space-filling. **(**b and c**)** Overlay of L1/*α*CT conformations in apo-ectodomain (brighter ribbons) and insulin-bound receptor fragment (darker tubes). Both chains of insulin are also rendered (yellow and black tubes). The displacement of *α*CT is indicated by a red arrow. Panel *c* in addition shows the steric overlap of the C-terminus of insulin B-chain (cyan tube) in free hormone with *α*CT position in the hormone-bound structure. The chain/domain coloring and labeling scheme is same as in Figure 1.

A number of studies [3,4,5,12,15,22,23,24,25,26,27,28,29,30,31,32,33,34,35,36,37,38,39,40,41,42,43,44,45,46] have suggested that insulin and IR each has two complementary binding epitopes known as “Site 1” and “Site 2” (Figure 2a): (a) both chains of insulin contribute residues to Site 1 (Gly^A1^, Ile^A2^, Val^A3^, Gln^A5^, Thr^A8^, Tyr^A19^, Asn^A21^, Val^B12^, Ty^r^^B16^, Phe^B24^, Phe^B25^, and Tyr^B26^) and Site 2 (Ser^A12^, Leu^A13^, Glu^A17^, His^B10^, Glu^B13^, Leu^B17^, and Val^B18^); and (b) in the receptor, L1 domain residues (Asp^12^, Ile^13^, Arg^14^, Asn^15^, Gln^34^, Leu^36^^,^ Leu^37^, Phe^39^, Glu^44^, Phe^64^, Tyr^67^, Phe^89^, Asn^90^, and Tyr^91^) and the *α*-chain C-terminus (*α*CT) residues (Phe^705^, Glu^706^, Asp^707^, Tyr^708^, Leu^709^, Asn^711^, Val^712^, Phe^714^, Pro^716^, and Arg^717^) contribute to Site 1, while residues in loops of the F1 domain (Lys^484^, Leu^552^, and Asp^591^) and the F2 domain (Ile^602^, Lys^616^, Asp^620^, and Pro^621^) likely contribute to Site 2. Moreover, productive engagement of insulin and IR (via Site 1 and Site 2) is a result of further conformational rearrangements in each due to plasticity in their structures. Particularly, the flexible C-terminus of the B-chain of insulin has been suggested to detach from rest of the insulin structure to expose its hydrophobic core to IR [47,48,49,50], which is potentially coupled with movement of the *α*CT peptide on IR [8,36,37,38,39,40,51,52,53,54,55,56,57,40,51]. These earlier observations are now strongly supported by recent (crystallographic) structural evidence on hormone-bound receptor fragments [58,59]. Importantly, alignment (based upon the L1 domain) of the apo-ectodomain structure (PDB code 3LOH) with a hormone-bound receptor structure (PDB code 3W11) unambiguously reveals the displacement of *α*CT (Figure 2b), and implies the detachment of the flexible C-terminus of insulin B-chain because *α*CT (in hormone-bound structure) occupies the position of this structural motif in free hormone (Figure 2c). Furthermore, we note that the exact displaced position of the C-terminus of the B-chain has now been resolved in latest structures by Menting *et al.* [59].

**Figure 3:**
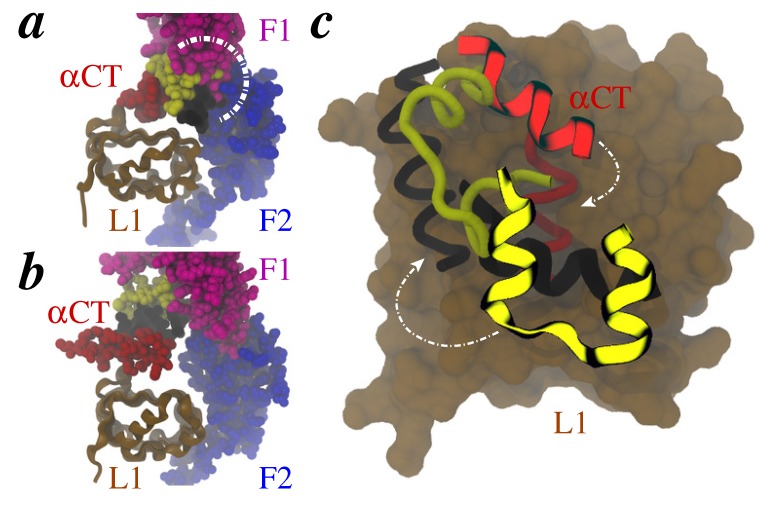
Insulin/IR complexes. **(**a**)** Alignment of insulin-bound structure of IR fragment (PDB code 3W11) with the apo-IR ectodomain (PDB code 3LOH). Insulin chains (A-chain, yellow; B-chain, black) and the F1 (magenta) and F2 (blue) domains of IR are rendered as space-filling, while the L1 domain (brown) is shown in a cartoon representation. Area of overlap between insulin residues and the F1/F2 loops is marked by a white arc. **(**b**)** Same view as in *a* from an all-atom structural model of insulin bound to IR ectodomain by Vashisth and Abrams [61]. Some residues of insulin are hidden behind the F1 and F2 domains. **(**c**) **Overlay of insulin/*α*CT on the L1 surface (brown) from the insulin-bound crystal structure (tubes) shown in *a*, and from the predicted model (cartoons) shown in *b*. White arrows indicate possible rotation of insulin/*α*CT (on the L1 surface) required to achieve crystal conformations starting with the predicted model conformations

While the recent structures of insulin bound to truncated receptors [58] have revealed unprecedented detail of registry (at Site 1) between hormone and IR, it is apparent that the conformation of hormone in these structures cannot be accommodated in the apo-IR ectodomain due to significant steric overlap [19,58] with the F1 and F2 domains (Figure 3a). This observation has led to two possibilities in that: (a) the F1-F2 pair has to undergo a conformational change [19] in the apo-IR ectodomain to accommodate insulin conformation observed in complexes with truncated receptors [58]; or (b) insulin/*α*CT may have to further rearrange in the ectodomain binding pocket if the F1-F2 pair was to maintain the conformation observed in the apo-IR ectodomain (PDB codes 2DTG and 3LOH). The second possibility can be discounted for various reasons: (1) a “see-saw” model [7] of negative-cooperativity in insulin binding [10] and a “harmonic-oscillator” model of receptor activation [60] require ligand-bound IR structure to be asymmetric unlike the symmetric conformation of the apo-IR ectodomain (Figure 1b); (b) the contacts of insulin with L1/*α*CT observed in truncated receptors [58,59] are consistent with a plethora of experimental evidence and therefore unlikely to change significantly; (c) a conformational change in the receptor is required to initiate signaling via activation of intracellular kinase modules [17]. In the absence of structural data on an insulin-bound IR ectodomain, the hypothesis concerning displacement of the F1-F2 pair (*vide supra*) can be tested using detailed atomistic simulation techniques as described below.

Building on our earlier work on all-atom structural models of ligand-bound IR [62] and IGF1R [63], we independently investigated the displacement of *α*CT on insulin binding to the IR ectodomain [61]. This work coincidently appeared around the publication of the crystal structures of insulin bound to truncated IR [58] and showed that (a) *α*CT is displaced on insulin binding; (b) the C-terminus of the insulin B-chain is likely positioned between L1 and *α*CT with Phe^B24^ and Tyr^B26^ oriented toward L1, while Phe^B25^ and Thr^B27^ toward *α*CT (Figure 5A in ref. [61]); and (c) the clockwise rotation of insulin/*α*CT together on the L1 surface leads to thermodynamically favorable (lower in free-energy) states. Importantly, there is no structural overlap between bound insulin and the F1-F2 pair in our previous model (Figure 3b). As a result, our predicted placement of insulin/*α*CT on the L1 surface is different from what is observed in a typical hormone-bound crystal structure (Figure 3c). Given that the IR ectodomain is flexible [62] and our predicted conformation of insulin/*α*CT is a metastable state, we speculated earlier that further structural rearrangements of insulin/*α*CT on the L1 surface are possible [61]. In fact, the overlay reveals that the crystal conformation of insulin/*α*CT may be achieved via a further clockwise rotation (white arrows in Figure 3c) of our predicted conformation on the L1 surface. In our earlier work [61], this would have additionally required displacement of the F1-F2 pair, a conformational change significantly challenging to observe in unbiased molecular dynamics (MD) simulations primarily due to difficulties in overcoming high free-energy barriers.

In this work, we judiciously combine our previous all-atom model [61] of insulin/IR∆*β* with information from crystallographically-resolved position of insulin/*α*CT [58] to directly show that crystal conformation of insulin can be achieved in the IR ectodomain, which in part is facilitated by a different placement of the F1-F2 pair. Interestingly, we find that such flexibility is present in the apo-IR ectodomain structure thereby reinforcing our previous suggestion that insulin recognition may be a ligand conformational-selection phenomenon [61]. Specifically, in a series of steps we carry out flexible fitting of our previous all-atom insulin/IR∆*β* model into simulated density maps created using crystal structures [58], and screening of receptor conformations from equilibration trajectories of the apo-IR ectodomain using our previously successful docking procedure [61,62,63]. The outcome is an all-atom model of insulin/IR∆*β* without any steric overlap of insulin and IR. Consistent with latest crystal structures [59], we also find that position of the C-terminus of insulin B-chain in our model is likely between L1 and *α*CT. We also outline key structural features of the IR ectodomain conformation in our new model when compared against the apo-IR ectodomain (PDB codes 2DTG and 3LOH).

## 2. Methods

### 2.1. Molecular Dynamics Flexible Fitting

Molecular Dynamics Flexible Fitting (MDFF) [64,65,66] is a simulation technique that incorporates information from an experimental or simulated electron microscopy (EM) map into an MD simulation for fitting an initial model of biomolecule in the target density map. Essentially, external steering forces arising from the potential encoded in the EM map are applied on top of the interatomic MD potential (force-field). Additional restraining forces can also be applied during fitting to prevent structural distortions and preserve secondary structure elements, to maintain stereochemistry [67] as well as the symmetry (if any) [68] of the biomolecule. A scaling factor *ξ >* 0 can be used to uniformly tune the effect of target map on all atoms. MDFF has been widely applied [69,70,71,72,73,74,75,76,77] to solve structures of large macromolecular complexes such as the ribosome. We have recently tested the effect of varying structural restraints as well as map resolution on fitting via MDFF, and provided recommendations for fitting proteins, nucleic acids, and their complexes [78,79].

In this work, we used MDFF to fit our previously proposed insulin/IR∆*β* structural model [61] into simulated density maps created using insulin/IR co-crystal structures [58]. Specifically, we created individual as well as combined density maps (at *∼*4 Å resolution and with a grid-spacing of 1 Å) of the L1 domain, insulin, and *α*CT using complex A (PDB code 3W11) of Menting *et al.* [58]. We created all density maps after alignment of the L1 domain of the crystal structure with the L1 domain of our previous model. For generating simulated density maps and associated map-derived potentials, we followed the previously established MDFF procedures [64,65,66].The starting model for fitting was built in the following way: first we extracted (from our previous model) the coordinates of L1/insulin/*α*CT, then we extended *α*CT to include residues 711–715 (after C*_α_*-alignment of residues 705–710 of the conformation of *α*CT in the crystal structure with the conformation of *α*CT in our previous model), following which, using our earlier Monte Carlo (MC) docking procedure [61,62,63] on equilibrated MD trajectories of the apo-IR ectodomain, we extensively searched for apo-IR conformations having no overlap with the target density map of L1/insulin/*α*CT, and finally we included coordinates of rest of the IR ectodomain from one of the lowest-energy states resulting from MC search. All hydrogens were included in our initial model as well as in the crystal structure from which maps were generated (*vide supra*). All simulations were carried out using NAMD [80,81] and the CHARMM force-field with the CMAP correction [82,83]. During fitting, all secondary structure elements were restrained with a force constant of *k* = 300 kcal mol*^−^*^1^ rad*^−^*^2^, and additional restraints were used to maintain the correct chirality of all chiral centers and *trans*-configurations of peptide bonds. The overall fitting procedure was optimized in three different steps: first by keeping insulin positionally-restrained, L1/*α*CT were allowed to flexibly fit using a combined density map of L1/*α*CT, then by keeping *α*CT positionally-restrained, fitting of insulin was carried out using a combined density map of L1/insulin, and finally L1/insulin/*α*CT were further fitted together (without restraints) using a combined density map of L1/insulin/*α*CT. The L1 density was eventually removed to further refine insulin/*α*CT, but it did not result in any significant improvement of model because all structural components are already correctly placed in the density after three-step procedure described above. The final model was energy-minimized and briefly MD-equilibrated.

## 3. Results and Discussion

### 3.1. MDFF Fitting

**Figure 4:**
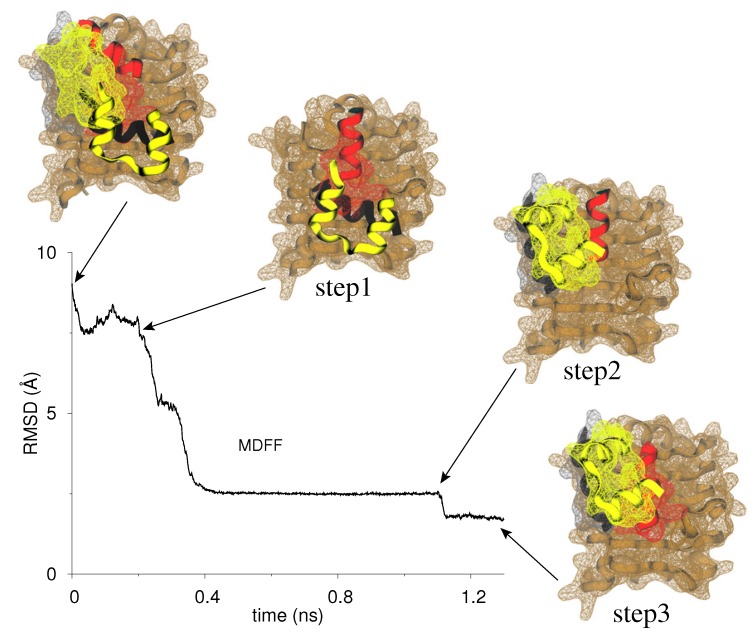
MDFF fitting of our initial insulin/IR∆*β* model into the target density maps of L1/insulin/*α*CT. The trace of C*_α_*-RMSD *vs.* simulation time (ns) for L1/insulin/*α*CT with respect to their final conformation in the crystal structure (PDB code 3W11) is shown. Snapshots are rendered at the beginning of MDFF run, and at the end of each step in the three-step procedure (see Methods). The coloring scheme for chains/domains is same as in Figure 3c. Maps are rendered as a wireframe mesh in same color as protein. Other domains of IR∆*β* are omitted for clarity.

In Figure 4, we show the root-mean-squared-deviation (RMSD) evolution of C*_α_*-atoms of L1/insulin/*α*CT relative to the crystal conformation (PDB code 3W11) during MDFF fitting. In step 1 (0 to 0.2 ns), we observe that nearly complete fitting of L1 is achieved while only a few N-terminal residues of *α*CT are fitted during this step which results in a straightened *α*CT conformation on the L1 surface. A major change in RMSD (*∼*6 Å) is observed during step 2 (0.2 to 1.1 ns) where both chains of insulin are successfully fitted. At the beginning of step 3, both L1 and insulin are already placed in their respective density maps but the C-terminus of *α*CT is still outside its target density, which is fitted in this final refinement step (1.1 to 1.3 ns). At the end of this three-step procedure, L1, insulin, and *α*CT are correctly placed in their target maps as indicated by a high final cross-correlation coefficient (CCC = 0.942) compared to its initial value (CCC = 0.745). The fitting process had no significant effect on the other domains of IR. The success of MDFF fitting suggests that the initial placement of L1/insulin/*α*CT observed in our previous model [61] is indeed a metastable state from which crystallographic conformation can be obtained. With regard to MDFF, it is important to point out that initially misplaced structure can still be fitted using MDFF in the target density map (due to underlying potential) albeit in a wrong orientation [78]. Therefore, it is noteworthy here that MDFF results in a correct placement of L1/insulin/*α*CT in the target density. In addition, as suggested in the introduction (Figure 3c), MDFF indeed proceeds by fitting insulin/*α*CT via a clockwise rotation (combined with displacement) on the L1 surface.

### 3.2. MDFF-Generated Model: Key Features

**Figure 5:**
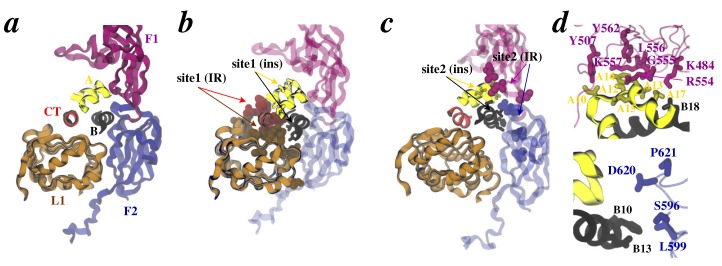
MDFF-generated model of insulin/IR∆*β*. **(**a**)** Final placement of insulin/*α*CT is shown in the MDFF-generated model of hormone/receptor complex. The hormone/receptor chains/domains are uniquely colored and labeled. Other domains of IR∆*β* are omitted for clarity. **(**b,c**)** Interacting residues at Site 1 and Site 2 of hormone (ins) and receptor (IR) are highlighted. Contacts at Site 1 are consistent with crystal structures [58], and no steric clash is present with suggested Site 2 residues [4,40] located in loops of the F1 and F2 domains. **(**d**) **Residue-residue contacts between insulin and IR at Site 2 are highlighted.

Salient features of our MDFF-generated model of insulin/IR∆*β* complex are shown in Figure 5. The overall orientation of insulin/*α*CT on the L1 surface is nearly identical to what is observed in the crystal structures of Menting *et al.* [58], while conformation of the F1-F2 pair is such that (*vide infra*) no overlap between insulin and the F1-F2 pair is observed (Figure 5a). The contacts at Site 1 between insulin and IR are also similar (Figure 5b) to the crystal structure (PDB code 3W11), while at site 2 (Figure 5c,d) we observe insulin residues (i) A12, A13, A14, A15, A17 in the vicinity of F1 residues R554, G555, L556, K557, and Y562; (ii) A10 and B18 near residues Y507 and K484 (F1), respectively; and (iii) B10 and B13 in the vicinity of S596, L599, D620, and P621 (F2). Other suggested Site 2 residues of the F2 domain (Ile^602^ and Lys^616^) are relatively distant from Site 2 residues on insulin. In addition, simultaneous contacts of insulin with Site 1 and Site 2 on IR, as observed here, is consistent with ligand cross-linking of receptor subunits [84] as well as a proposed sequential model [85] of association of insulin with IR (via Site 1 and 2).

**Figure 6:**
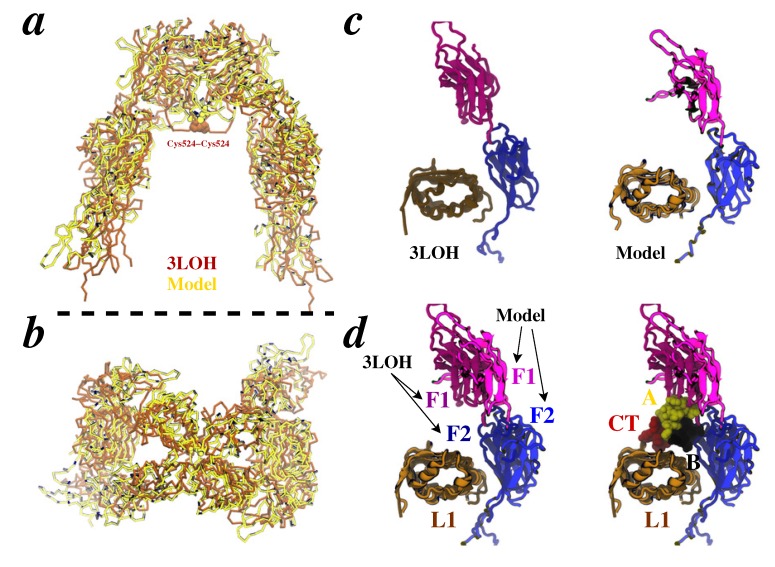
Comparison of apo-IR∆*β* and MDFF-generated model. **(**a,b**)** Overlay of apo-IR crystal structure (brown; PDB code 3LOH) and our MDFF-generated model (yellow). Front (*a*) and top (*b*) views are rendered. The dotted black line represents the location of membrane. Insulin and *α*CT are omitted. **(**c,d**)** Displacement of the F1-F2 pair is highlighted. Conformation of the L1-F1-F2 fragment from the apo-IR crystal structure (left panel in *c*) and our model (right panel in *c*) of insulin/IR∆*β* complex; structures were aligned based upon C*α*-atoms of the L1 domain. Overlay of panels displayed in *c* is also shown without (left panel in *d*) and with (right panel in *d*) insulin/*α*CT (space-filling), respectively.

We further compare the IR∆*β* conformation in our model with that of apo-IR∆*β* (Figure 6a,b). We quantify conformational differences by using our previously defined [61,62] collective-variable conformational metrics (based upon the centers-of-mass of domains) of IR∆*β* such as interdomain hinge-angles. Because apo-IR∆*β* crystal structure is symmetric (Figure 1b), all pairs of interdomain hinge angles are identical in the IR homodimer: the F1-F2 hinges are at 160.80°, the F2-F3 hinges are at 174.59°, the L1-L2 hinges are at 90.33°, and the apex L2-F1 hinges are at 81.5°. On insulin-bound side of IR∆*β* in our model, we observe that the F1-F2, F2-F3, and L1-L2 hinges are contracted by 9°, 13°, and 7° (in comparison to apo-IR∆*β*), respectively, while the L2-F1 hinge at the apex of IR∆*β* is open by 6°. On the other (unliganded) binding pocket, the F1-F2 and F2-F3 hinges are nearly identical to apo-IR∆*β*, while the L1-L2 and apex L2-F1 hinges are contracted by 14° and 3°, respectively. The closing of F1-F2 and L1-L2 hinges coupled with the opening of the apical L2-F1 hinge on one side of the IR homodimer leads to one binding pocket more open than the other; an asymmetric flexibility mechanism we have earlier observed in apo-IR∆*β* [62]. Consistent with a “see-saw” model [7] of negative-cooperativity [10] and a “harmonic-oscillator” model of receptor activation [60], the conformation of IR∆*β* in our model is asymmetric. The exact conformations of the L1-F1-F2 motifs from our model and the apo-IR∆*β* crystal structure are also depicted (Figure 6c,d), and show that closing of the F1-F2 hinge leads to a displaced conformation of the F1-F2 pair that can accommodate insulin in crystallographic position without any steric clashes.

Finally, we attempted to model the conformation of the flexible C-terminus of the B-chain of insulin in our MDFF-generated model. Aligning the conformation of insulin from our previous model of this complex [61] with that in the crystal structure reveals that the C-terminus of insulin B-chain is likely located between L1 and the C-terminus of *α*CT with minor steric clashes of residues B29/B30 of insulin with L1 (cyan tube in Figure 6a). Therefore, we carried out a short MC-refinement of the C-terminus of insulin B-chain by selecting low-energy states from an ensemble of conformations of this motif previously generated by enhanced sampling and free-energy methods [61]. One such conformation of the C-terminus of insulin B-chain was incorporated in the MDFF-generated model, and the resulting complex was briefly equilibrated in explicit water. The overlay of many conformations of L1/insulin/*α*CT from this short MD-run is shown in Figure 6*b*, where C-terminus of insulin B-chain appears to fluctuate between L1 and *α*CT. These conformations are qualitatively similar to recently-resolved [59] position of the C-terminus of insulin B-chain.

**Figure 7:**
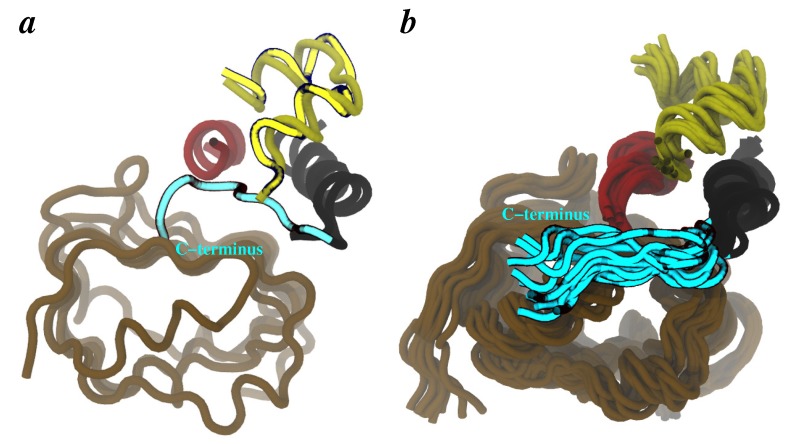
Conformation of the C-terminus of insulin B-chain. **(**a**)** Insulin conformation from our previous [61] structural model of insulin/IR∆*β*(brighter tubes) is overlayed on the insulin conformation in the hormone-bound crystal structure (darker tubes, PDB code 3W11). Alignment of insulin from both structures suggests new location of the separated C-terminus of insulin B-chain (cyan tube). **(**b**)** MD-equilibrated ensemble of L1/insulin/*α*CT configurations from MDFF-generated model after incorporation of the C-terminus of the B-chain of insulin (cyan). Other domains of IR∆*β* are not shown for clarity.

## 4. Conclusions

In this work, we have presented an all-atom structural model of an insulin/IR∆*β* complex, where insulin conformation is consistent with hormone-bound structures of truncated receptors. Importantly, we show that crystallographic conformation of insulin can be accommodated in the IR ectodomain conformation in our model without steric clashes. On the ligand-bound side of IR in our model, a different placement (relative to apo-IR∆*β*) of the first two type-III fibronectin domains (F1 and F2) of receptor supports the previously suggested hypothesis [19,58] that a conformational change in these two domains is needed for insulin docking in the IR ectodomain. We also observe simultaneous contacts at Site 1 and Site 2 between insulin and IR, and suggest that the C-terminus of insulin B-chain is likely located between L1 and *α*CT. This work also demonstrates how structural information from experiments can be combined with simulation techniques to further refine all-atom models.

The atomic coordinates of the model presented in this work can be obtained from the author on request.
